# The Association between Material Deprivation and Avoidable Mortality in Lisbon, Portugal

**DOI:** 10.3390/ijerph17228517

**Published:** 2020-11-17

**Authors:** Claudia Costa, Angela Freitas, Ricardo Almendra, Paula Santana

**Affiliations:** 1 Centre of Studies in Geography and Spatial Planning, University of Coimbra, 3004-530 Coimbra, Portugal; angelafreitas30@gmail.com (A.F.); ricardoalmendra85@gmail.com (R.A.); paulasantana@uc.pt (P.S.); 2 Department of Geography and Tourism, University of Coimbra, 3004-530 Coimbra, Portugal

**Keywords:** inequalities, area deprivation, avoidable deaths, premature mortality, preventable deaths, amenable deaths due to healthcare, HIV/AIDS, suicide, urban health, cities, geography

## Abstract

There is considerable evidence pointing to the existence of a socioeconomic gradient in mortality, which tends to be steeper in urban areas. Similar to other European cities, Lisbon is far from homogeneous since considerable geographical inequalities exist between the more advantaged and the more deprived neighborhoods. The main goals of this study are to describe the geographical pattern of premature deaths (before 65 years old), avoidable deaths (preventable and amenable to healthcare) and cause-specific mortality (HIV/AIDS and suicide) in Lisbon, at the lower administrative level (civil parish, in Portuguese: Freguesia), and analyze the statistical association between mortality risk and deprivation, before (1999–2003) and during the economic crisis (2008–2012). Smoothed Standardized Mortality Ratios (sSMR) and Relative Risk (RR) with 95% credible intervals were calculated to identify the association between mortality and deprivation. The analysis of the geographical distribution of cause-specific mortality reveals that civil parishes with high sSMR in the first period continued to present higher mortality rates in the second. Moreover, a significant statistical association was found between all the causes of death and deprivation, except suicide. These findings contribute to understanding how social conditions influence health outcomes and can offer insights about potential policy directions for local government.

## 1. Introduction

Inequalities in health are a persistent challenge worldwide, as there are considerable variations associated with socioeconomic factors across and within countries [1,2,3], as well as within cities [4,5]. However, most studies focus on country-to-country comparisons, which may not be appropriate for informing local policymaking because data can mask intra-urban differences [6].

Differences regarding the demographic, economic, and environmental characteristics, the different level of access to housing, amenities, and services, and to other societal challenges impacting cities explain the presence of intra-urban inequalities [7,8]. Considering previous studies on European cities, socioeconomic inequalities in health continue to be of greater magnitude in more urbanized communities, where vulnerable people are clustered in impoverished neighborhoods and deprived areas, usually in the suburbs of the city or in inner city areas [9,10,11]. Costa et al. [12] observed that municipalities performing worse in health determinants also present worse health outcomes. Samoli et al. [13] discovered that, in poor urban areas, poorer air quality is usually observed. Both studies agree that it is important to identify these areas to support local action [12,13] because poor circumstances might be modifiable by policies at the local level [14], and acting in these places enables the greatest impact on reducing inequalities [15,16]. Therefore, tackling social inequalities in health is a major priority [17].

There is considerable evidence pointing to the existence of a marked geographical and socioeconomic gradient in mortality in urban areas [18], namely between municipalities in the same region or metropolitan area [12,19] and even at the smaller area level [9,20,21]. Thus, the small area level is considered the most favourable geographical scale for avoiding the ecological bias created by heterogeneity within the area, and for detecting spatial mortality trends that may not be apparent in larger geographical areas. However, the number of cases within the small area is often reduced. The application of Bayesian models like the hierarchical BYM model, developed by Besag, York, and Moliè [22], accounts for the spatial auto-correlation of individuals by pooling information from neighboring regions, overcoming this limitation related to observations with small outcome counts [23,24].

Material deprivation is a major health determinant that refers to the lack of fundamental needs, which are context- and time-specific [25]. According to Townsend [26], it is multidimensional and includes a wide range of variables (e.g., education, employment, and housing conditions) that can be translated through a composite indicator. These indexes, that started being developed in the 1970s, and are now available in almost all the European Countries, contribute to a better understanding of spatial inequalities in health since evidence suggests the effect of material deprivation on health inequalities [3].

Material deprivation is one of the most suitable measures to understand mortality disparities within urban areas [24,27,28]. Thus, urbanization exacerbates the adverse impacts of material deprivation on mortality [29]. Previous ecological studies have found an association between a material deprivation composite index and total mortality [9], premature deaths [28,30], avoidable mortality amenable to healthcare [23], diabetes [31], infectious diseases [31], cancer [32], dementia [33], suicide [24], ischemic heart disease [34], cancer [35], cerebrovascular disease [36], chronic liver disease [29], and traffic injuries [21]. In the context of crisis and austerity, these associations tend to increase, as vulnerable populations appear to be more affected by the negative consequences of austerity measures [19,37,38]. Nonetheless, studies to date regarding total mortality in several European cities revealed that the 2008 economic crisis did not change this pattern in the short term [39].

By focusing on the lower administrative level of the City of Lisbon, Portugal’s capital, and considering the period both prior to (1999–2003), and during the economic crisis (2008–2012), this analysis aims to: (i) characterize the geographical pattern of mortality, and (ii) analyze the statistical association between mortality and material deprivation. Three groups of causes of death, considered avoidable and highly associated with deprivation, have been selected: (1) deaths before age 65 (premature mortality); (2) deaths that could have been avoided through effective public health and primary preventive interventions (preventable deaths); (3) deaths that could have been avoided by prompt and efficient health care services (amenable deaths due to healthcare). Deaths from HIV/AIDS were also analyzed due to high mortality rates in the Lisbon Metropolitan Area [40]. Finally, suicide and self-intended harm were also included in this study because their increase is considered one of the first signs of an economic crisis [24].

## 2. Materials and Methods

### 2.1. Study Area

In addition to being the capital of Portugal, Lisbon is the political, economic, social, and cultural heart of the Lisbon Metropolitan Area, where over a quarter of the Portuguese population lives. Specifically, the municipality of Lisbon is home to almost 5% (509,515 inhabitants) of the Portuguese residents, presenting a population density of 5047 hab./km^2^. Still, the number of city users (resident and non-resident population working or studying in the city) increases by more than 70% due to the movements of those who commute.

More than a quarter of Lisbon City inhabitants have higher education and a competitive wage level, as compared with other cities: the average monthly earning is almost 50% higher when compared with the rest of Portugal [41]. Similar to other southern European cities, the population is becoming older, particularly within the city centre: the population aged 65 or over represents 24% while the young are 12% of the population. The improvements to the transportation network and increase in purchasing power during the 1970s and 1990s allowed many families to move to the surrounding municipalities, relegating the city centre to older citizens (24%) and immigrants (11%) [42]. Recently, tourism has changed this pattern, with many buildings being renovated and adapted for local touristic accommodation, resulting in displacement and gentrification in several historic neighborhoods [43,44].

The municipality of Lisbon is subdivided into civil parishes (in Portuguese: Freguesias), the lowest administrative unit of local government in Portugal, and the most appropriate level to capture inequalities and enable intra-city analysis. Civil parishes share many responsibilities with municipalities, mostly related to the fulfilment of the needs of local communities (e.g., socio-economic development, spatial planning, public transport, housing, social care, culture, environment) [45].

### 2.2. Design, Source of Information and Indicators

An ecological study was performed on trends based on two periods, one prior to the economic crisis (1999–2003) and one during the crisis (2008–2012). Data on mortality, population, and socioeconomic conditions were provided by the National Statistics Institute (in Portuguese: Instituto Nacional de Estatística—INE) for 42 civil parishes in Lisbon. Due to data confidentiality, mortality from 12 civil parishes from the city centre, with an average population of 1833 inhabitants in 2001, were aggregated into a larger geographic unit covering 3 km^2^ and a total population of 12,822 inhabitants.

A total of five causes of death were analyzed: premature mortality, preventable deaths, amenable deaths due to healthcare, HIV/AIDS, and suicide (Table 1). Notice that both preventable deaths and amenable deaths due to healthcare are based on the list of avoidable causes of death defined by the official statistical office of the European Union (EUROSTAT), which also specifies an age limit of 74 years old for almost all avoidable causes of death [46]. Data on mortality was stratified by age groups and aggregated for the periods of analysis. The resident population from 2001 and 2011 was stratified and aggregated in the same way as the mortality data.

The material deprivation indicator was based on the approach adopted by Santana et al. [19,31,47]. This composite metric takes into account three dimensions: education, employment, and housing conditions. The indicators selected to translate these dimensions (from the 2001 Census) were: (i) illiteracy rate (population over the age of 10 unable to read and write); (ii) unemployment rate (unemployment in the labour force); (iii) substandard housing rate (houses with no toilet). Table 2 provides some descriptive information about these three indicators, as well as population. Based on the Carstairs and Morris method [48], the indicators considered in this index were standardized (using the *z*-score method) so that each indicator has a weighted mean of zero and a variance of one, and exerted the same influence upon the final result. As this measure is dimensionless and arbitrarily fixed, we introduce it into the model in terciles, with t1 representing the civil parishes with lower deprivation and t3 those with higher deprivation.

### 2.3. Analysis

The Standardized Mortality Ratio (SMR) was calculated for the total population of the city of Lisbon.

Due to the population size and the low number of deaths, we used the hierarchical Bayesian model introduced by Besag, York, and Mollié (BYM) to obtain smoothed SMR (sSMR) and the probability of excess risk [22]. This approach helps to generate smoothed estimates while still providing a clear spatial pattern, mitigating potential bias [20]. BYM controls for the spatial dependency between geographical areas, which means that adjacent neighbors are more likely to have a similar mortality level than distant areas, along with the non-spatial variability. Thus, this model takes the spatial and the heterogeneous random effects into account to provide smoothed estimates and a clear spatial pattern [21,23,31].

For each cause of death, the sSMR was calculated using the following model:

(1) $$O_{i}\sim Poisson\left(E_{i}\theta_{i}\right)\;Log\left(\theta_{i}\right)=\alpha +S_{i}+H_{I}$$

where, for each small area *i*, *O_i_* is the number of observed deaths for a particular cause of death in the small area *i*, *E_i_* is the expected number of deaths (of each cause) in the small area *i* and *θ*_i_, the relative risk for each area and cause of death. *α* represents the intercept, *S_i_* the spatial random effect, and *H_i_* the heterogeneous (non-spatial) effect. The expected number of deaths in each area was determined by indirect standardization, using the Lisbon population in 2001 and 2011 (multiplied by the number of years in each sample period: 5 years), and using mortality rates by age and cause of death as reference.

We also calculated the likelihood of mortality excess risk based on sSMR (sSMR > 100). The value 100 reflects the Lisbon standard; that is, the mortality ratio if there were no variations between civil parishes.

In addition, to analyze the trend regarding the association with material deprivation, we fitted an ecological regression model that incorporates this composite indicator as an explanatory variable. The regression was formulated as follows:

Log(θ_i_) = β_1_ + β_2_ X_2 i_ + β_3_ X_3 i_ + S_i_ + H_i_ (2)

where exp(β_2_) (respectively, exp(β_3_)) denotes the relative risk of mortality in the areas included in the second tercile, X_2 i_ (respectively, third tercile, X_3 i_) group with respect to those included in the first tercile deprivation group (X_1 i_).

The relative risk (RR) was obtained based on their posterior means, along with the corresponding 95% credible intervals (95% CI). An RR was considered significantly higher or lower than 1 if its 95% CI did not include 1. The posterior distributions were obtained using the “Integrated Nested Laplace Approximation” (INLA) method [49]. An intrinsic conditional autoregressive prior distribution (ICAR) was assigned to the spatial effect, while the heterogeneous effect is represented using independent normal distribution [22]. A half-normal distribution with mean 0 and precision 0.0001 was assigned to the standard deviations σ_s_ and σ_h_. A vague prior distribution was assigned to the parameters β_1_, β_2_, and β_3_. Notice that the parametrization of the model may be complex and influence the outcome of the analysis, so a sensitivity analysis was performed in order to best select the parameters of the model. Other authors use this methodology to perform disease mapping and socioeconomic inequalities in mortality at the small area level [9,20,50]. These models were developed using the INLA library (version 3.0.1) recommended by Rue et al. [49] and the R statistical package.

### 2.4. Limitations of the Study

The findings of this study must be viewed with certain drawbacks in mind. First, it is important to note that the data used to build the material deprivation index is from 2001, and the deprivation of areas may have changed over time. This option was already taken by other researchers [24,39] considering the probability that the ranking of areas by socioeconomic deprivation would remain the same.

Second, due to the need to maintain confidentiality at statistical level, the access to mortality data was only possible with aggregated data from 12 parishes in the city centre. As a consequence, the situation of these parishes has been subsumed in the analysis as it may obscure the kind of statistical association between variables. Furthermore, we only have access to the deceased person’s last place of residence and we do not know how long the person had been living there.

Third, according to the EUROSTAT’ list of avoidable causes of death, some diseases and conditions are included in both amenable and preventable deaths, since certain conditions are considered as both treatable and preventable (examples are ischemic heart diseases and diabetes) [46]. Therefore, the rates cannot be summed up without duplication.

Fourth, the amenable deaths due to healthcare and preventable deaths indicators have been changing since the beginning of the period, with causes of death being included or excluded due to changes in treatments and implementation of policies [46]. Therefore, the selection of the causes of death are, to some extent, arbitrary (Plug et al., 2012) and specific to a time. Moreover, setting the age limit at 75 years excludes a large number of potentially treatable or preventable deaths in older people, reducing the potential of detecting significant change.

Fifth, while our research contains a pattern overview of 13 years, it would be useful to examine longer trends in order to be able to assess changes over a larger number of years and even after the economic crisis. However, in 2012, civil parishes were reorganized and their number within each municipality decreased. Lisbon reduced its number of civil parishes from 53 to 24 which, in some cases, meant redrawing new administrative boundaries. Therefore, it is impossible to compare data before 2013 with data from 2013 onwards.

Finally, in terms of analysis, there are two key issues: (i) indirect age standardization of mortality data reduces the effect of heterogeneity of age distributions, which does not completely eliminate the effect of the population’s age structure; (ii) the presence of statistical association must be carefully interpreted in terms of causality [19]. Therefore, cause-specific mortality maps can only be used to point out possible issues at the small area level, which then have to be researched with more detailed knowledge and more contextual evidence of the interaction between health determinants and health outcomes.

## 3. Results

Between the first and the second period, the cause-specific mortality in Lisbon municipality decreased, with HIV-AIDS and amenable deaths due to healthcare registering the largest decrease (less 67% and 35%, respectively) (Table 3). In contrast, the number of deaths by suicide increased slightly in the same period (more 10%).

**Figure 1:**
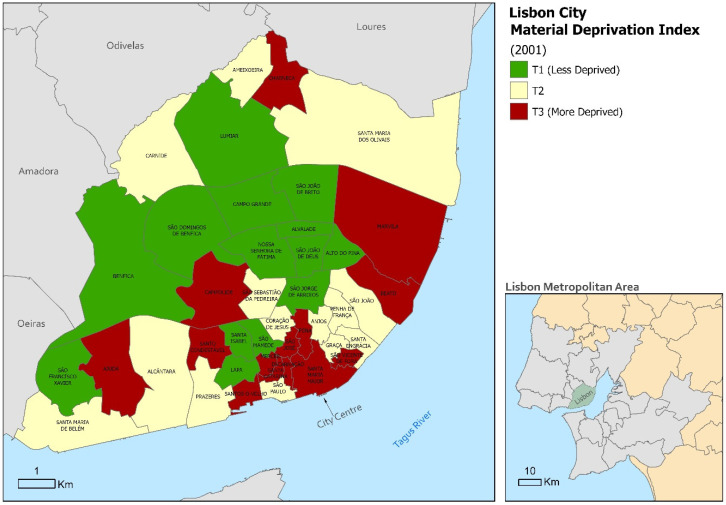
Material Deprivation across Lisbon civil parishes, 2001. Source: based on Census data from the National Statistics Institute.Source: based on Census data from National Statistics Institute.

Figure 1 reveals the geographical pattern of material deprivation across the municipality of Lisbon. The pattern of material deprivation is characterized by better conditions in the northern and western civil parishes, and worse conditions in the civil parishes located along the Tagus River, in the historic centre, and the eastern part of the municipality (around the port area).

**Figure 2:**
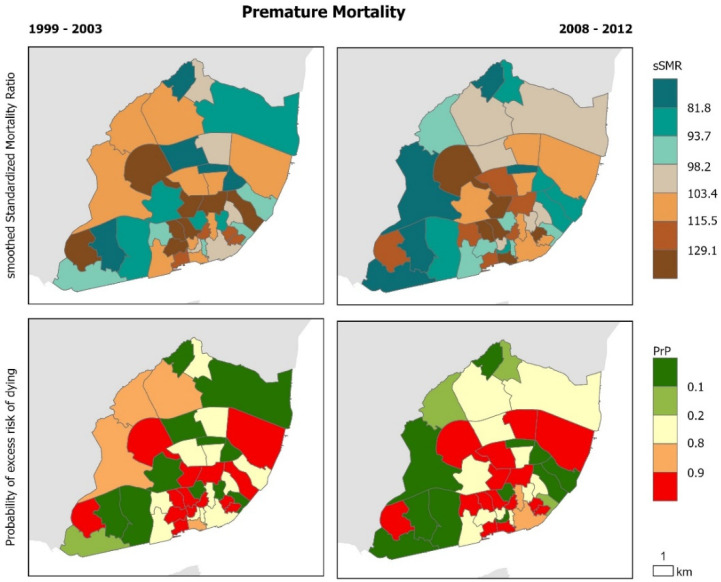
Premature Mortality in Lisbon Municipality. Smooth Standardized Mortality Ratio (sSMR) and probability of excess risk of dying (PrP), 1999–2003 and 2008–2012.

**Figure 3:**
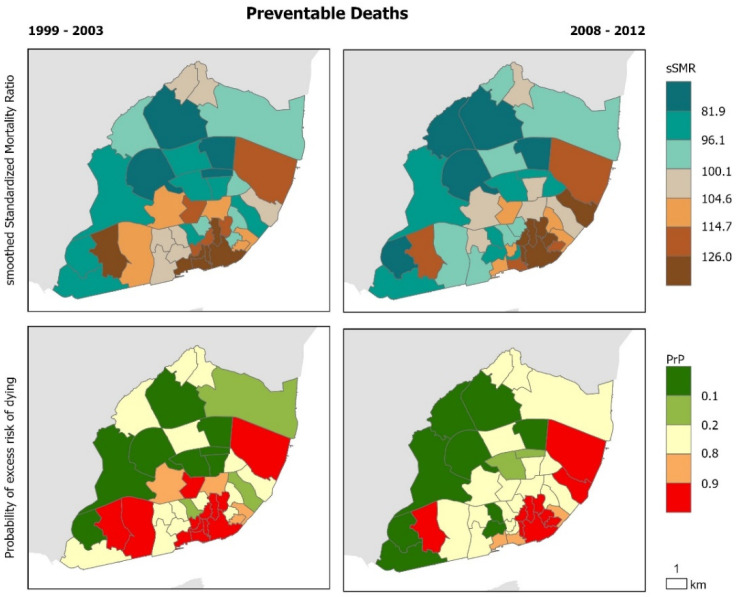
Preventable deaths in Lisbon Municipality. Smooth Standardized Mortality Ratio (sSMR) and probability of excess risk of dying (PrP), 1999–2003 and 2008–2012.

**Figure 4:**
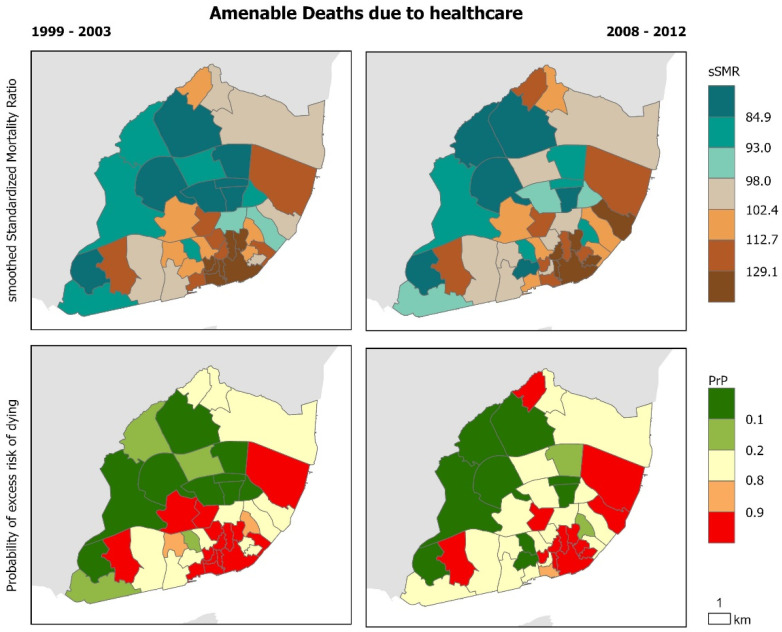
Amenable deaths due to healthcare in Lisbon Municipality. Smooth Standardized Mortality Ratio (sSMR) and probability of excess risk of dying (PrP), 1999–2003 and 2008–2012.

**Figure 5:**
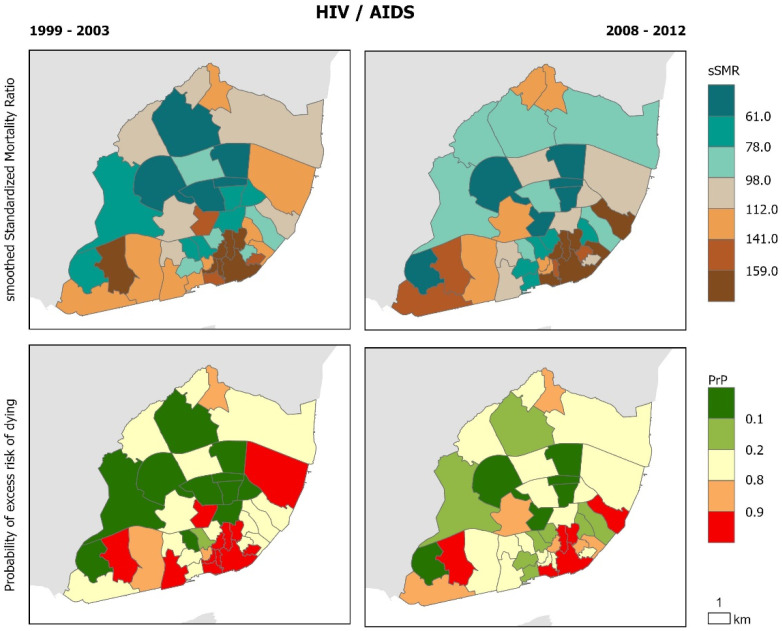
HIV/AIDS Mortality in Lisbon Municipality. Smooth Standardized Mortality Ratio (sSMR) and probability of excess risk of dying (PrP), 1999–2003 and 2008–2012.

**Figure 6:**
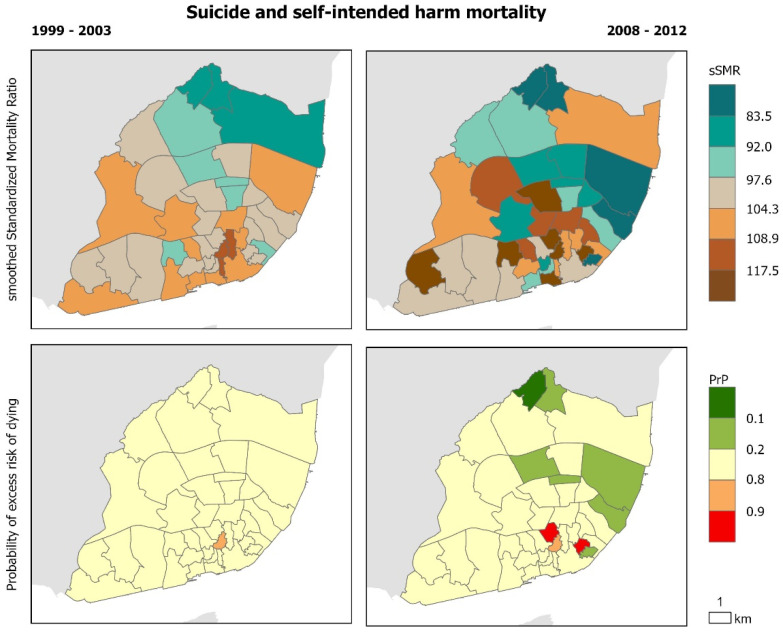
Suicide and self-intended harm Mortality in Lisbon Municipality. Smooth Standardized Mortality Ratio (sSMR) and probability of excess risk of dying (PrP), 1999–2003 and 2008–2012.

The geographical distribution of cause-specific mortality confirms that there are intra-urban variations that remain similar before and during the crisis (Figure 2, Figure 3, Figure 4, Figure 5 and Figure 6). Suicide sSMR is the cause of death with a higher level of pattern change between the two periods (Figure 6). Results also provide evidence that the geographical pattern is similar: in almost all causes of death there is a northwest–southeast gradient of mortality characterized by higher mortality in the civil parishes of the historic centre and along the Tagus River. These parishes also correspond to those with a significantly higher probability of excess risk of dying (90% probability that the sSMR is higher than the pattern of the City of Lisbon) from the analyzed causes of death.

**Figure 7:**
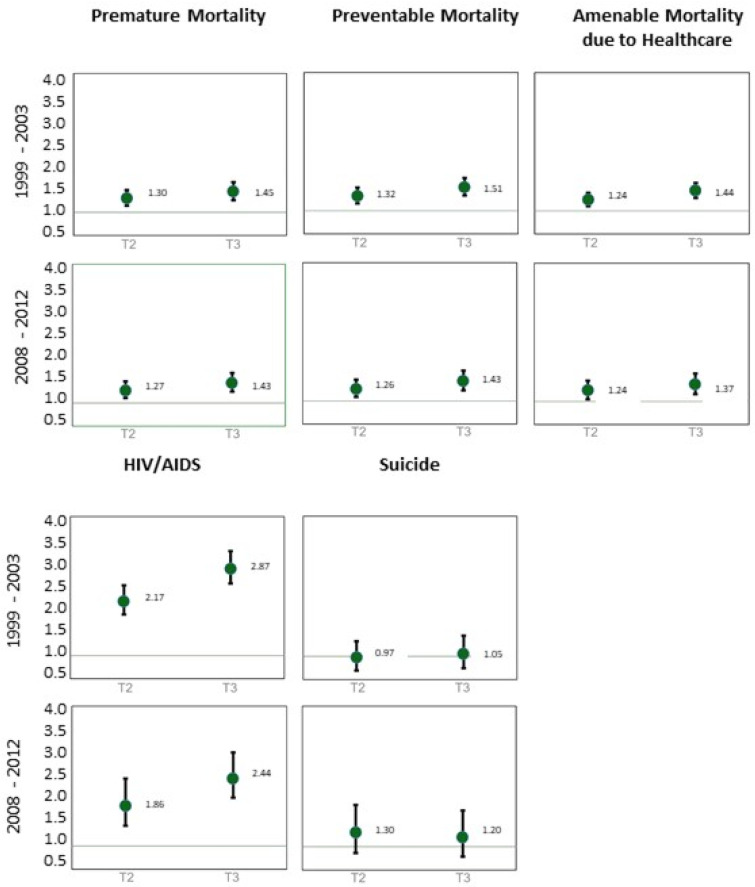
Relative Risk (RR) and 95% Credible Intervals (95%CI) between material deprivation (the second and third tercile (most deprived) compared to the first tercile (less deprived)) and mortality by cause of death in Lisbon.

Figure 7 presents the outcomes of the ecological regression that provided evidence of the statistical association between material deprivation, in terciles, and cause-specific sSMR. All causes of death display a positive significant statistical association: the population living in the most deprived areas are at greater risk of dying from the analyzed causes. Suicide is the only exception. HIV/AIDS was the cause of death revealing a higher risk associated with deprivation in both periods: in the last period, the mortality risk was 144% higher in more deprived civil parishes than in their less deprived counterparts. During the crisis, the association between material deprivation and mortality decreased for premature, preventable, and amenable to healthcare deaths.

## 4. Discussion

This study provides evidence that: (i) mortality is unevenly distributed across the municipality of Lisbon; (ii) there is a strong association between mortality and material deprivation; (iii) during the economic crisis there was a decrease in the association between material deprivation and cause-specific mortality; (iv) suicide is an exception since it is the only cause of death that increased between periods, and it does not reveal an association with material deprivation.

Important intra-urban variation was found, as shown by the geographic pattern of mortality across the municipality and the association with material deprivation. In both periods, there is a strong association between higher levels of material deprivation (third tercile) and higher risk of mortality. This was identified for all causes of death analyzed, except for suicide. This association is particularly evident in the civil parishes located in the historic centre of Lisbon. This part of the city is generally characterised by a high proportion of elderly residents and migrants living in old and degraded housing, along with other vulnerable population groups affected by poor social and economic conditions [31,42]. Previous studies revealed that social and economic deprivation are the major drivers for Lisbon’s health inequalities, requiring interventions that go beyond the health sector [18]. Thus, previous research identified considerable geographical inequalities between the more advantaged and deprived civil parishes in Lisbon: along the 8 km distance of the Metro’s Blue Line, there is a 6-year difference in average life expectancy [51].

The bulk of the causes of death analyzed in this research showed a decrease during the economic downturn. There is no agreement as to whether mortality rates rise or decrease during times of financial hardship [52,53,54,55]. Our results are similar to others, revealing that mortality during the crisis seems to follow the downward trend observed prior to the crisis [39,56] which might be masking an increase for certain socio-economic groups more adversely affected by the economic crisis [57,58]. Thus, our study focuses on the short-term impact of the economic recession, but the consequences might need a longer period of time to be determined [24,39,54,58].

Civil parishes with high material deprivation reveal higher risk of mortality due to HIV/AIDS: 44% more than those living in civil parishes with lower material deprivation. This might explain the association identified for premature, preventable, and treatable deaths since this cause of death is also included in these groups. Previous studies already reported the high incidence of cases in Lisbon, especially among immigrants [40]. The low utilization of primary healthcare services and poor access to specialist care by migrants in Lisbon is one of the reasons, with consequences for early diagnosis and timely access to treatment [59,60]. The low systematic access to healthcare could be explained by job insecurity, legal, cultural, and linguistic barriers, and lack of knowledge about access to healthcare [61].

As the nation’s capital, Lisbon has good geographical access to healthcare services and high numbers of health professionals. However, the risk of dying from an amenable cause of death due to the healthcare services is not equal across the municipality. The RR reveals a significant statistical association with material deprivation: civil parishes in the worst tercile reveal a 37% higher risk of dying from this group of causes of death when compared with the less deprived ones. Previous studies have also shown that deprived areas are at increased risk of mortality due to worse access to, and poorer quality of, healthcare services [23]. Although the last austerity period was characterized by drastic cuts in public budgets, strict regulation of public expenditure, a reduction in patient travel funding, and a rise in consumer charges [37], the number of amenable deaths associated with healthcare services decreased during the economic downturn, as found in other studies [62]. Still, the impact from those measures may have long-term effects on mortality amenable to the healthcare.

When comparing the period prior to and during the economic crisis, suicide mortality was the only cause where the number of deaths increased. Thus, several studies identified an association between suicide and material deprivation in Portugal [19,63], and an increase during the recent economic crisis [19,24,64,65]. This exception might be explained by the geographical scale of this study, the financial instability experienced in Portugal [19,66], and the effect of the economic crisis on suicidal behaviour, which was transversal to all socioeconomic groups of society [24]. During the last austerity period, Portugal faced repercussions in terms of unemployment, emigration, purchasing power, and inability to afford housing costs [37,38,67], which affected both privileged and deprived areas [24] and, consequently, their access to healthcare [68]. On the one hand, the private sector lost many clients that were not able to afford their services due to loss of income; on the other, the public sector suffered blind cuts in expenditure, increases in co-payments, and reduced state subsidies for some treatments. As a result, some patients started to miss mental healthcare appointments, cut down medication, and only accessed the health system for acute situations [69,70,71].

### 4.1. Policy Implications

This study reveals that mortality increases as material deprivation does, which adds support to the findings of previous studies focusing on Portugal and the Lisbon Metropolitan Area [19,20,23,31,72,73]. More deprived civil parishes accumulate multiple factors impacting population health negatively (e.g., low levels of income and education, unemployment, and poor housing conditions). Public policies addressing the economic and social inequalities among groups and geographical areas have the potential to mitigate and reduce the associated risk of mortality at an upstream level. Those interventions might include: (i) the creation of job opportunities and job-search support; (ii) improvement in housing quality for poor and deprived families; (iii) the provision and promotion of access to decent and affordable housing; (iv) the prevention of school drop-out, from pre-school up to lower secondary education, along with lifelong learning opportunities and training; (v) promotion of social support to low-income and vulnerable families, among others [74]. Moreover, interventions addressing individual health behaviour and access to healthcare are also extremely important. Examples include: (i) health literacy campaigns designed for specific vulnerable groups, such as sex workers, teenagers, and immigrants; (ii) access to early cancer screening; (iii) improving access to quality services adequate to the demographic and cultural characteristics of their users (e.g., immigrants); (iv) providing a place for support, counselling, and information able to advocate for the vulnerable groups’ interests and empowering their communities.

So far, Lisbon Municipality already implemented some programmes that might help to achieve equity between parishes like: (1) the Entrepreneurship and Employability Program for young people (PEEJ) and the Municipal Program for Social Economy and Promotion of Employability in Lisbon (PMESPEL), which aims to support job-search and creation; (2) the Local Housing Program (PLS), the Municipal Lease Subsidy to House Rents Program (SMA) and the Affordable Rent Program, which aims to help families to improve their houses or to be able to afford a house in the municipality; (3) The Social Development Plan 2017–2020 (PDS), which aims to support education and training activities and promote social support to vulnerable families; (4) the Municipal Plan for the Integration of Immigrants in Lisbon 2015–2017 (PMIL), which aims to promote health literacy on healthy-behaviours and access to healthcare dedicated to the immigrant population; (5) the Health Development and Quality of Life Plan for the City of Lisbon 2017–2020 (PDSQVBEL), which aims to produce health literacy campaigns on healthy behaviours designed to vulnerable groups; (6) the Health + Next Program, which aims to increase accessibility to screening programs. The potential of these policies to promote health equity was evaluated by Freitas and colleagues [75].

### 4.2. Strengths of the Study

Studies looking at the roots of health inequalities are vital to improving health outcomes; namely, reducing avoidable deaths.

First, this study contributes to improving local knowledge of the main issues affecting health outcomes at the local level, informing local decision-makers on where to act and what to act on. By examining the association between material deprivation and mortality, this type of analysis can inform local city health profiles and plans relative to the areas affected by higher rates of mortality and the associated risk factors. This can encourage intersectoral action amongst different public services from educational, social, housing, and health sectors to collaborate and act together in these areas. These results were presented to the Lisbon Municipality, contributing to inform the Health Plan for Quality of Life and Wellbeing of the City of Lisbon 2019–2020 [76]. Additionally, they integrated the evidence base for conducting local workshops with a panel of stakeholders, including decision-makers, to identify critical situations and inform local policies aimed at reducing health inequities [77].

Second, as recent studies have shown, people living in deprived areas are more likely to be affected by most of the known risk factors for COVID-19, either directly or indirectly. Factors such as occupational activity, housing conditions, health-related behaviours, literacy level and the presence of comorbidities are intertwined, interactive, and cumulative in those areas [78,79]. COVID-19 highlights and exacerbates health inequalities [80,81]. It is important to compile evidence and evaluate these patterns in order to develop an appropriate and tailored approach for future COVID-19 waves or pandemics.

Focusing on the city of Lisbon, this study provides a picture of existent inequalities at the local level, identifying the civil parishes where people disproportionally die from avoidable causes of death associated with their poor socioeconomic status and living conditions. These neighborhoods represent a current concern for local government and health authorities due to their high risk of COVID-19. To address them, it is crucial to integrate responses tailored to the local context and oriented to health determinants.

## 5. Conclusions

This study adds greater understanding of health and its determinants in the city of Lisbon. The local and geographical-based analysis of cause-specific mortality and associated deprivation is a stepping-stone in the identification of “urban areas under warning” and essential to redirect local government to address locally the socioeconomic determinants of health inequities. Thus, bottom-up approaches based on local data and knowledge are of considerable relevance for equity-based policies, thus enabling cities to achieve its Sustainable Development Goals (SDGs), namely Goal 3 and 10, respectively “Good health and wellbeing” and “Reduced Inequalities”.

## Figures and Tables

**Table 1:** Groups of causes of death and specific causes, by age group and ICD10.

Mortality	Age Group	ICD10 Cause of Death
Premature Mortality	0–65	all
Preventable deaths	0–49	E10–E14
	0–74	A15–A19, B90, B15–B19, B942, C00–C14, C15, C16, C18–C21, C22, C33–C34, C43, C50, C53, F10, F11–F16, F18–F19, I20–I25, J40–J44, J47, K70, K73–K74
	All	B20–B24, V01–V99, Y85, W20–W64, W75–X39, X50–X59, Y86, X60–X84, Y870, X85–Y09, Y871
Amenable deaths due to healthcare	0–49	E10–E14
	0–74	A15–A19, B90, B15–B19, B942, C18–C21, C43, C50, C53, C67, C73, C81–C85, C91–C95, I20–I25, I60–I69, J02, J09–J18, J45–J46, K25–K28, N00–N29, Q00–Q99
	All	B20–B24, P00–P96
HIV/AIDS	All	B20–B24
Suicide and self-intended harm Mortality	10+	X60–X84, Y87.0

**Table 2:** Descriptive characteristics of the Lisbon Municipality’ parishes—2001.

Indicator	Minimum	Average	Standard Deviation	Maximum
Population (nº)	3182	13,444	10,694	46,410
Illiteracy rate (%)	2.1	6.1	2.1	12.9
Unemployment rate (%)	4.6	7.2	1.4	11.3
Substandard housing rate (%)	0	0.7	0.5	1.9

Source: based on Census data from National Statistics Institute.

**Table 3:** Description of mortality data in Lisbon, by cause of death: number of deaths, crude mortality rate (MR) per 100,000 inhabitants, and share of total mortality.

	Total Deaths	Premature Deaths	Amenable Deaths due to Healthcare	Preventable Deaths	HIV/AIDS	Suicide
1999/2003	Number of deaths	11,411	7423	8588	285	276
	Crude MR	404.2	262.9	304.2	10.1	9.8
	% total deaths	27.8	18.1	20.9	2.5	0.7
2008/2012	Number of deaths	7840	4234	5567	95	304
	Crude MR	286.3	154.6	203.3	3.5	11.1
	% total deaths	21.9	11.9	15.6	1.2	0.9

Source: based on Mortality registers from the National Statistics Institute.
